# Late-Onset Cerebral Autosomal Dominant Arteriopathy With Subcortical Infarcts and Leukoencephalopathy (CADASIL) Presenting With Abnormal MRI Findings and Migraine: A Case Report

**DOI:** 10.7759/cureus.115259

**Published:** 2026-08-26

**Authors:** Fifi Gho, Christopher Seet

**Affiliations:** 1 Neurology, National Neuroscience Institute, Singapore, SGP

**Keywords:** cadasil, cerebral autosomal dominant arteriopathy with subcortical infarcts and leukoencephalopathy (cadasil), late-onset cadasil, migraine disorder, periventricular hyperintensities, stroke

## Abstract

Cerebral autosomal dominant arteriopathy with subcortical infarcts and leukoencephalopathy (CADASIL) is a hereditary small-vessel disease caused by mutations in the *NOTCH3* gene. It typically presents in mid-adulthood with migraine with aura, recurrent ischemic strokes or transient ischemic attacks, and progressive cognitive decline. Late-onset presentations are uncommon and may pose diagnostic challenges due to atypical clinical features. We report a case of late-onset CADASIL in a patient who initially presented with intermittent migraine and abnormal MRI findings. Neuroimaging revealed extensive white matter hyperintensities involving the periventricular and deep white matter, as well as the external capsules and the corpus callosum, raising suspicion of CADASIL. Genetic testing confirmed a pathogenic variant in the *NOTCH3* gene. This case highlights the importance of considering CADASIL in older patients with unexplained MRI abnormalities and migraine, even in the absence of classic early symptoms. Early diagnosis is essential for appropriate management, family screening, and avoidance of unnecessary interventions.

## Introduction

Cerebral autosomal dominant arteriopathy with subcortical infarcts and leukoencephalopathy (CADASIL) is a hereditary cause of stroke and vascular dementia in adults. It is an autosomal dominant condition caused by mutations in the *NOTCH3* gene on chromosome 19, leading to degeneration of vascular smooth muscle cells and progressive small vessel disease. Common presentations include transient ischemic attacks (TIAs) and strokes, cognitive impairment, psychiatric disturbance, and migraine [1,2].

The disease typically presents between the third and fifth decades of life, with migraine with aura often being the earliest manifestation. However, late-onset CADASIL is rare and often under-recognized. Such cases may lack the characteristic clinical progression and can be mistaken for sporadic ischemic cerebral small vessel disease.

Patients with CADASIL in East Asian populations, who have a higher proportion of the *NOTCH3* R544C variant, tend to present at a later age and have a reduced prevalence of migraine with aura. In a Taiwanese study, patients with this pathogenic variant presented later, at a mean age of 54.1 years [3].

Neuroimaging plays a critical role in diagnosis, with hallmark findings including symmetrical white matter hyperintensities, particularly involving the external capsules and anterior temporal poles [4]. Molecular genetic testing for pathogenic *NOTCH3* variants provides definitive confirmation of the diagnosis.

We present a case of late-onset CADASIL identified through abnormal MRI findings in a patient with intermittent migraine, emphasizing diagnostic considerations and clinical implications.

## Case presentation

A 60-year-old Singaporean Chinese woman presented to the neurology clinic following an abnormal brain MRI that demonstrated extensive bilateral white matter hyperintensities. The MRI was performed as part of the workup for intermittent headaches suggestive of episodic migraine without aura. The headaches were infrequent, unilateral, pulsating, and occasionally associated with nausea, but without photophobia, phonophobia, or aura. She had no significant past medical history of stroke, TIA, cognitive decline, or psychiatric illness. She had a brother who developed dementia at the age of 78, characterized by amnesia and executive dysfunction with disinhibition.

The patient’s neurological examination was unremarkable, with no focal neurological deficits observed. The workup for secondary causes of white matter disease was unremarkable. She had no history of diabetes mellitus, hyperlipidemia, or hypertension. Screening for antiphospholipid syndrome, systemic autoimmune disease, and vasculitis was negative.

**Figure 1:**
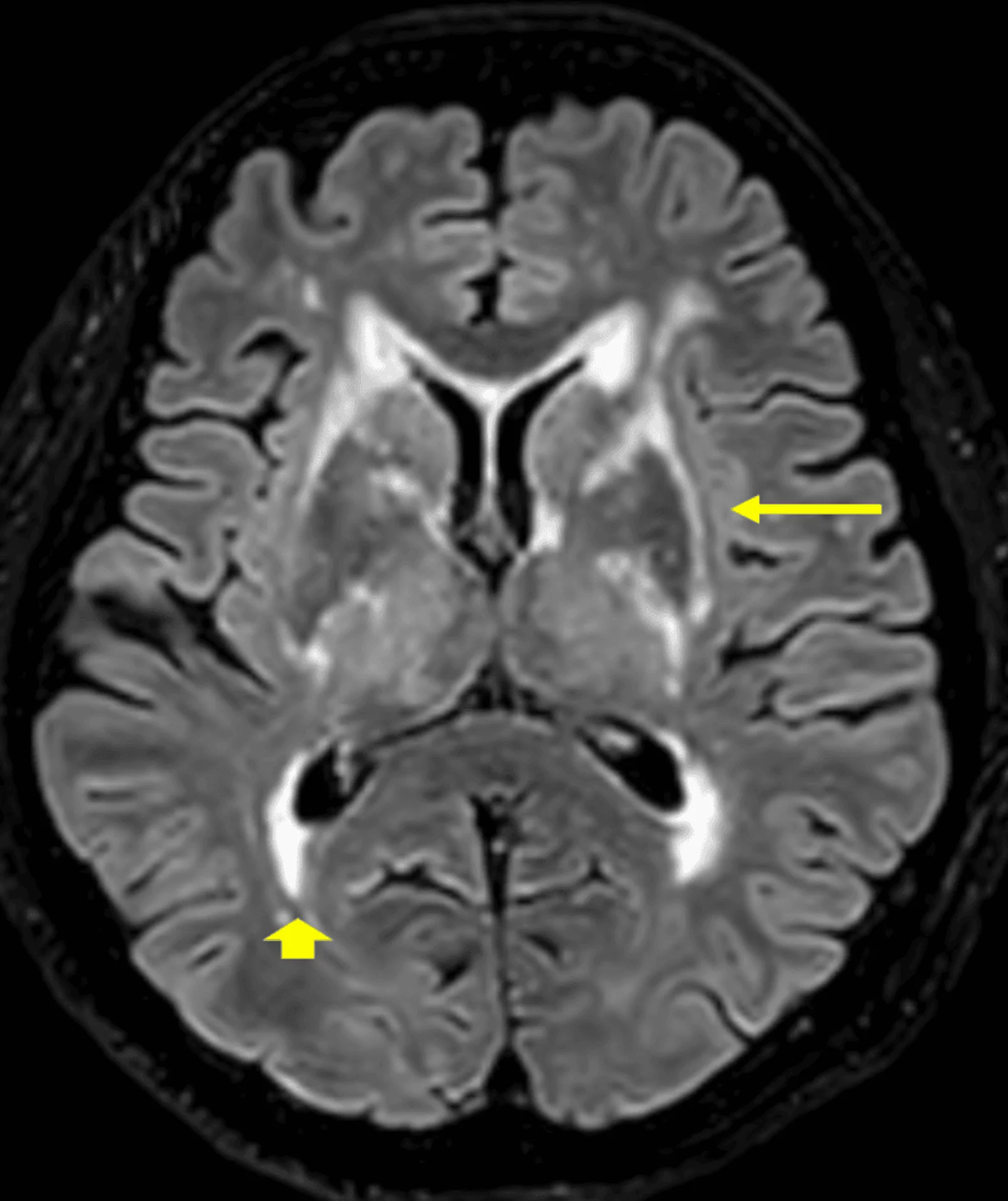
Axial brain MRI fluid-attenuated inversion recovery sequence at the level of the basal ganglia. Brain MRI showing extensive fluid-attenuated inversion recovery hyperintensities in the periventricular (short yellow arrow) and deep white matter, including bilateral external capsules (long yellow arrow).

**Figure 2:**
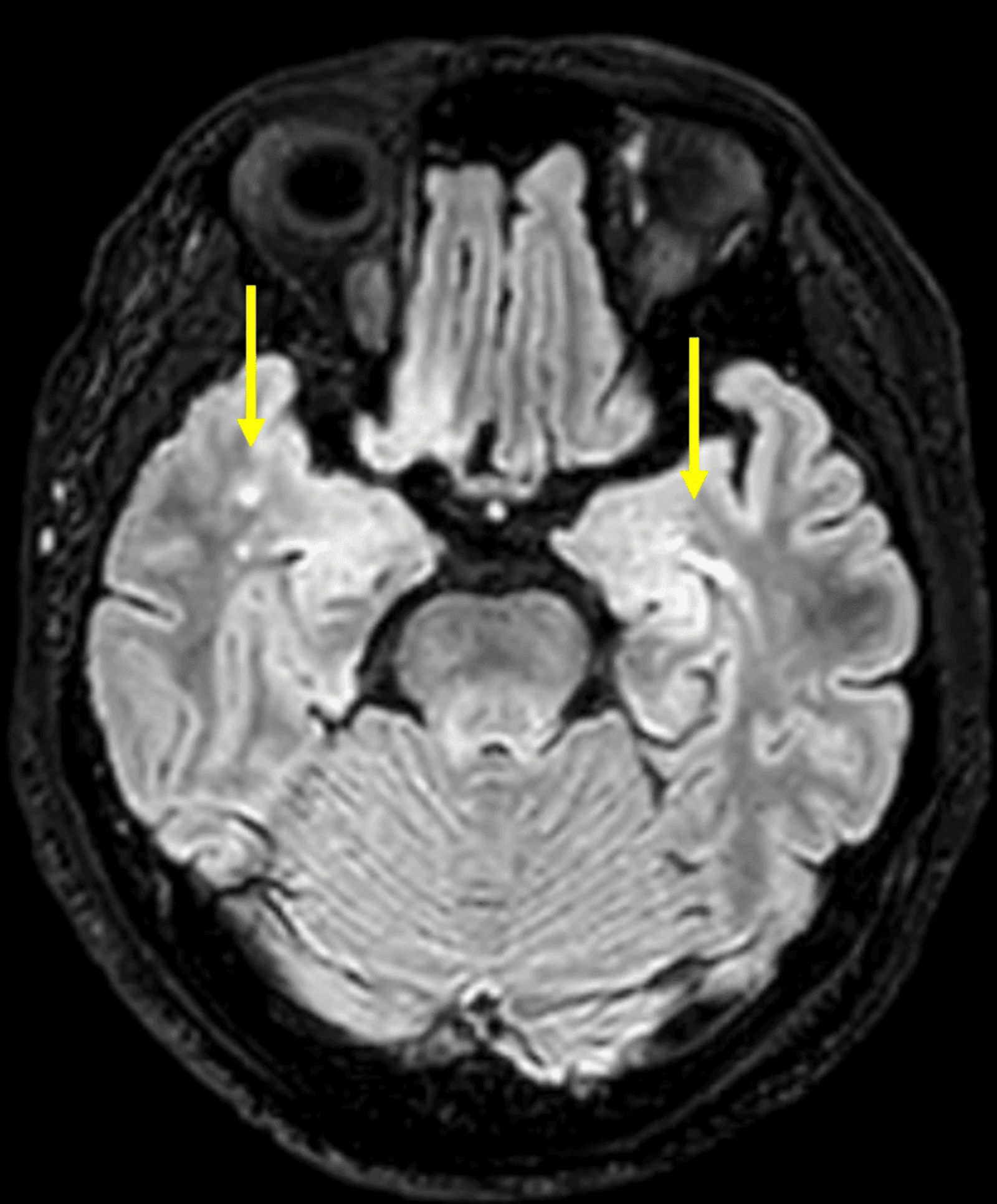
Axial brain MRI fluid-attenuated inversion recovery sequence at the level of anterior temporal poles. Brain MRI showing subtle fluid-attenuated inversion recovery hyperintensities in bilateral anterior temporal poles (yellow arrows).

**Figure 3:**
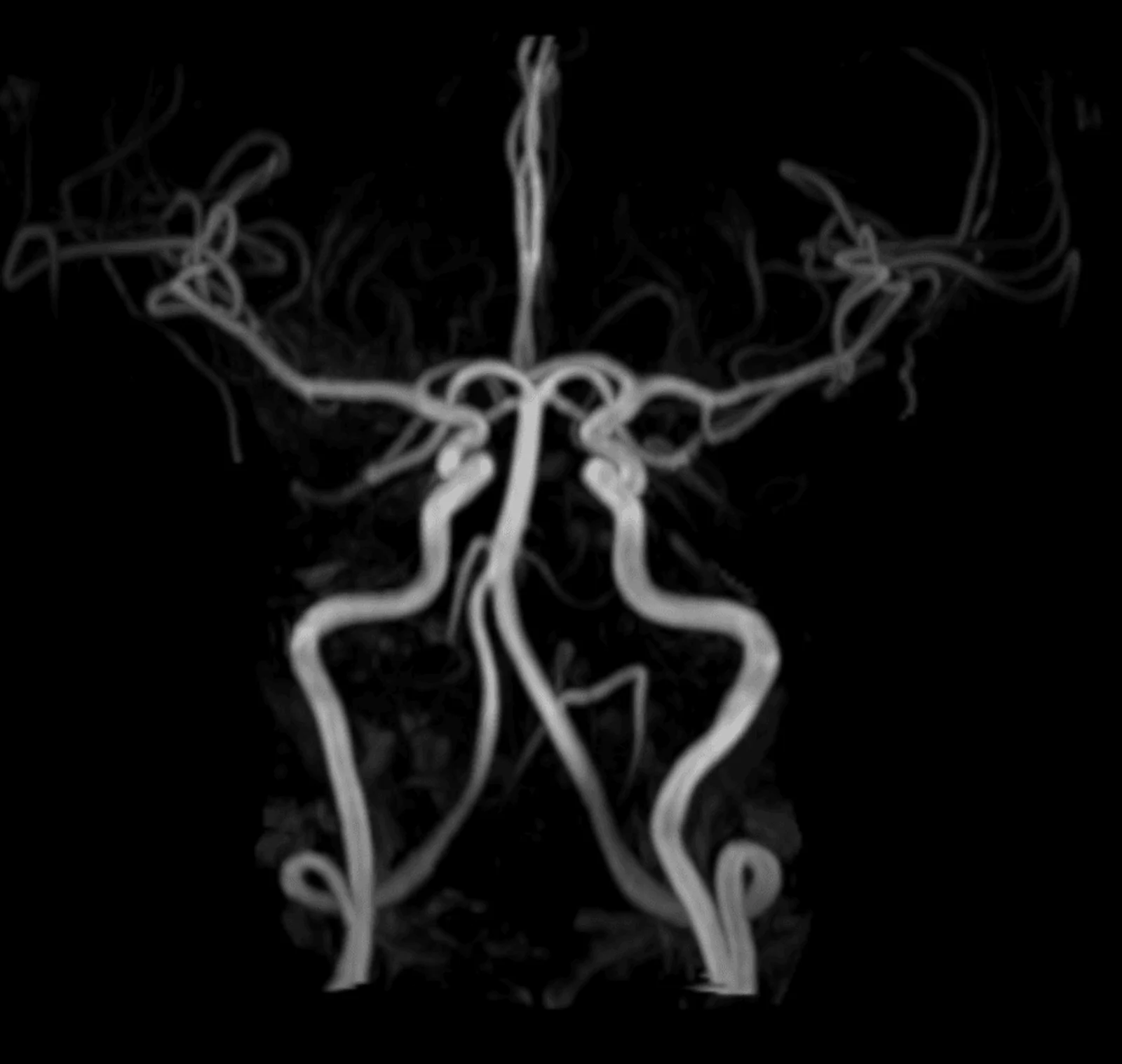
Brain magnetic resonance angiography time-of-flight sequence. Intracranial magnetic resonance angiography shows that the vessels are patent. There is no vascular beading to suggest vasculitis.

Four months after her initial presentation, a repeat MRI of the brain with contrast and magnetic resonance angiography (MRA) was performed. The MRI revealed extensive, fairly symmetrical patchy and confluent hyperintensities on T2-weighted and fluid-attenuated inversion recovery (FLAIR) sequences in the periventricular and deep white matter, including bilateral external capsules (Figure 1) and subtle FLAIR hyperintensities in bilateral anterior temporal poles (Figure 2). The Fazekas score was 3 [5]. A small chronic infarct was noted in the left side of the corpus callosum. There was no vascular beading to suggest vasculitis (Figure 3) and no pathological enhancement. On the gradient echo (GRE) sequence, no cerebral microbleeds were observed. Given the characteristic MRI findings, CADASIL was suspected.

Soon after the repeat MRI, she developed transient dysarthria and was diagnosed with TIA. The repeat MRI of the brain showed no acute infarct and was otherwise stable. No cerebral microbleeds or other major hemorrhagic risk factors were identified. Following the TIA, she underwent vascular risk factor modification and was initiated on single antiplatelet therapy (aspirin 100 mg once daily) and high-intensity statin therapy (atorvastatin 40 mg once daily) for secondary stroke prevention.

Further evaluation included genetic testing using an adult-onset leukodystrophy and leukoencephalopathy panel, which identified the pathogenic *NOTCH3* variant, c.1630C>T (p.Arg544Cys) (R544C), confirming the diagnosis. She was counseled regarding the genetic nature of the disease, and some of her family members underwent *NOTCH3* gene testing. The clinical course and key diagnostic events are summarized in Table 1.

**Table 1:** Timeline of events. TIA = transient ischemic attack; CADASIL = cerebral autosomal dominant arteriopathy with subcortical infarcts and leukoencephalopathy

Timeline	Events
Month 0	Presented with episodic migraine without aura and abnormal brain MRI findings
Month 4	Repeat brain MRI was performed. She subsequently developed TIA shortly after the MRI. Antiplatelet therapy and statin therapy was initiated
Month 6	CADASIL was formally diagnosed following identification of a pathogenic *NOTCH3* variant
Month 18	No further recurrence of TIA or stroke after initiation of antiplatelet and statins. No evidence of cognitive decline

The patient was subsequently followed up in clinic. There was no recurrence of stroke or TIA after the initiation of aspirin and statins. There was no evidence of cognitive decline during follow-up visits.

## Discussion

CADASIL is characterized by progressive degeneration of cerebral small vessels due to accumulation of granular osmiophilic material [6]. While migraine with aura is often the earliest manifestation, late-onset cases may present atypically.

In East Asian populations, where the exon 11 R544C variant is relatively common, patients with CADASIL tend to present at a later age [3]. They also show a lower proportion of moderate or severe white matter lesions in the anterior temporal poles, a higher frequency of cognitive dysfunction, and a lower prevalence of migraine [1,3,7] compared with Caucasian populations, in whom pathogenic variants most commonly occur in exons 3 and 4 [8].

Late-onset CADASIL has been increasingly recognized, often associated with milder phenotypes or delayed progression [9]. In this case, the patient had symptom onset at age 60, presenting with episodic migraine without aura and subtle involvement of the anterior temporal poles. She carried the pathogenic *NOTCH3* R544C variant, which is relatively common in East Asian populations. The absence of previous neurological events further contributed to the diagnostic challenge.

MRI findings remain an important diagnostic clue. The involvement of the anterior temporal lobes and external capsules is highly suggestive of CADASIL and helps differentiate it from other causes of cerebral small vessel disease such as hypertensive arteriopathy. Unlike sporadic cerebral small vessel disease, patients with CADASIL often demonstrate corpus callosum and anterior temporal involvement, as seen in this patient.

Genetic testing for pathogenic *NOTCH3* variants provides definitive molecular confirmation of CADASIL and enables screening of at-risk family members.

Management of CADASIL is largely supportive, as no disease-modifying therapy currently exists. Treatment focuses on controlling vascular risk factors, managing migraine, and preventing stroke. The benefits of antiplatelet therapy in patients with CADASIL are uncertain, given the lack of randomized controlled trials, and individualized risk-benefit assessment is essential. CADASIL patients with hypertension have a higher prevalence of cerebral infarction and intracranial hemorrhage, and a significantly greater number of cerebral microbleeds, than those without hypertension [10-13].

In this patient, the presence of migraine without aura, together with the delayed presentation, contributed to the atypical clinical picture and diagnostic delay. Her family history of dementia further supported the consideration of hereditary cerebral small vessel disease. Her MRI findings were consistent with the reported phenotype associated with the R544C variant.

In terms of management, single antiplatelet therapy was initiated following the TIA. She had neither cerebral microbleeds nor hypertension. However, the absence of cerebral microbleeds does not eliminate the risk of intracranial hemorrhage. The risks and benefits of antithrombotic therapy should therefore be considered on an individual basis. Routine use of dual antiplatelet therapy or anticoagulation should generally be avoided unless there is a compelling independent indication [14], particularly in patients with cerebral microbleeds, hypertension, or other hemorrhagic risk factors.

This case underscores the importance of considering CADASIL in older patients with unexplained white matter changes, even when classical clinical features are absent. Accurate and timely diagnosis allows for better counseling and avoidance of inappropriate investigations and management.

## Conclusions

Late-onset CADASIL is an uncommon but important differential diagnosis in patients presenting with migraine and abnormal MRI findings. This case highlights that CADASIL can present beyond the typical age range and may lack classical clinical features. Recognition of characteristic MRI patterns, particularly involvement of the anterior temporal lobes and external capsules, should raise strong suspicion for CADASIL but is not diagnostic on its own and should prompt further genetic evaluation in the appropriate clinical context. Clinicians should maintain a high index of suspicion for CADASIL in older patients with unexplained MRI changes, particularly when family history suggests hereditary vascular disease. Accurate and timely diagnosis facilitates appropriate management and genetic counseling for affected individuals and their families.
